# Enhanced Readout from Spatial Interference Fringes in a Point-Source Cold Atom Inertial Sensor

**DOI:** 10.3390/s23115071

**Published:** 2023-05-25

**Authors:** Jing Wang, Junze Tong, Wenbin Xie, Ziqian Wang, Yafei Feng, Xiaolong Wang

**Affiliations:** Key Laboratory of Quantum Precision Measurement of Zhejiang Province, College of Science, Zhejiang University of Technology, Hangzhou 310023, China; 1112002006@zjut.edu.cn (J.W.); 2112009035@zjut.edu.cn (J.T.); 2112109113@zjut.edu.cn (W.X.); 221122090101@zjut.edu.cn (Z.W.); 2112109050@zjut.edu.cn (Y.F.)

**Keywords:** atom interferometry, principal component analysis, cold atom inertial sensor

## Abstract

When the initial size of an atom cloud in a cold atom interferometer is negligible compared to its size after free expansion, the interferometer is approximated to a point-source interferometer and is sensitive to rotational movements by introducing an additional phase shear in the interference sequence. This sensitivity on rotation enables a vertical atom-fountain interferometer to measure angular velocity in addition to gravitational acceleration, which it is conventionally used to measure. The accuracy and precision of the angular velocity measurement depends on proper extraction of frequency and phase from spatial interference patterns detected via the imaging of the atom cloud, which is usually affected by various systematic biases and noise. To improve the measurement, a pre-fitting process based on principal component analysis is applied to the recorded raw images. The contrast of interference patterns are enhanced by 7–12 dB when the processing is present, which leads to an enhancement in the precision of angular velocity measurements from 6.3 μrad/s to 3.3 μrad/s. This technique is applicable in various instruments that involve precise extraction of frequency and phase from a spatial interference pattern.

## 1. Introduction

Analogous to optical interference, wave-particle duality allows for interference of microscopic particles. Unlike neutrons and electrons, atoms possess distinctive internal energy-level structures and properties such as discrete mass, magnetic moments, and polarizations, making atomic interference capable of revealing information on physical fields in detail. Atomic interferometry holds significant importance in the domain of precision measurements. Additionally, atom interferometers have been successfully utilized for measuring gravitational acceleration [1,2], fine structure constant [3], angular velocity [4,5], etc., and can be used to verify the equivalence principle and general relativity [6]. In recent years, they have been proposed for applications in gravitational wave detection for better precision than long-path optical interferometers [7].

An atom point-source interferometer (APSI) is a new type of atomic inertial sensor capable of measuring both gravitational acceleration and angular velocity [8]. These instruments read the interference phase and contrast in a single measurement cycle, avoiding the need for compensating for velocity-dependent phase shifts. This feature helps to improve the sampling rate and signal-to-noise ratio. For the past decade, APSIs have been applied in atomic gyroscopes [9], gravimeters [10], and gravity gradiometers [11].

Following the point-source model [12], the spatial position and velocity of an atom in a cloud of ultra-cold atoms are coupled after the cloud is tightly focused. Therefore, the velocity-dependent phase is directly reflected in the spatial distribution of an atom cloud after free expansion in vacuum, and phase information of APSIs can be obtained by detecting the phase of spatial interference fringes.

To accurately extract the spatial frequency of the interference patterns of an atom cloud in such an interferometer, we have applied specific methods based on principal component analysis (PCA) [13] in processing raw atomic fluorescence images. This technique effectively filters out influences of atomic envelops, stray background light, and other detection noises. The signal-to-noise ratio of the spatial fringes are improved by 5–12 dB, and the fringe contrast is also significantly improved. Compared with direct fitting of the fringes from a raw image, the measurement precision of spatial periods is improved by one order of magnitude, leading to about twice-higher precision of angular velocity measurements out of the same atom interferometer. This technique can be combined with a variety of techniques to obtain an increase in measurement performance, for example with LMT for higher sensitivity [14].

## 2. Theoretical Methods

### 2.1. Phase Shear of Atom Point Source Interferometry

In an atom interferometer, the interference phase shift rising from the gravitational acceleration g and angular velocity of Earth rotation Ω are expressed as:

(1) $$\Delta \Phi_{g}=-k_{eff}\cdot gT^{2},$$

(2) $$\Delta \Phi_{\Omega }=2k_{eff}\cdot (\Omega \times v)T^{2},$$

where $k_{eff}$ is the effective wave-vector of stimulated Raman transition, T is the time interval between Raman pulses, and v is initial velocity of the atom under investigation.

In point-source approximation, the atom cloud in the interferometer is focused to a size much smaller compared with its final size after free expansion. When the initial cloud before expansion is regarded as a volume-less point with a certain velocity distribution, the influence of an atom’s initial position on its final displacement is neglected and its displacement r=vt is proportional to its initial velocity v during the evolution time t of free expansion.

The displacement–velocity coupling in the point-source model makes the APSI sensitive to rotations, even in a single axis interferometer that is conventionally used in gravitational acceleration measurements. Here is demonstrated the measurement of angular velocity $\Omega_{x}$ along the horizontal x-axis as an example. The velocity of the atom cloud along the y-axis is denoted $v_{y}$, so its displacement at moment t along the y-axis can be expressed as $y=v_{y}t_{d}$ according to the point-source model. The spatial fringe frequency caused by mirror rotation can be described by phase gradient $\kappa_{x}=\partial \Delta \Phi_{\Omega }/\partial y=2k_{eff}\Omega_{x}T^{2}/t_{d}$, where $t_{d}$ is the free expansion time of the atom cloud. The angular velocity is thus obtained by measuring the spatial fringe frequency, which makes the principle of the APSI gyroscope.

**Figure 1:**
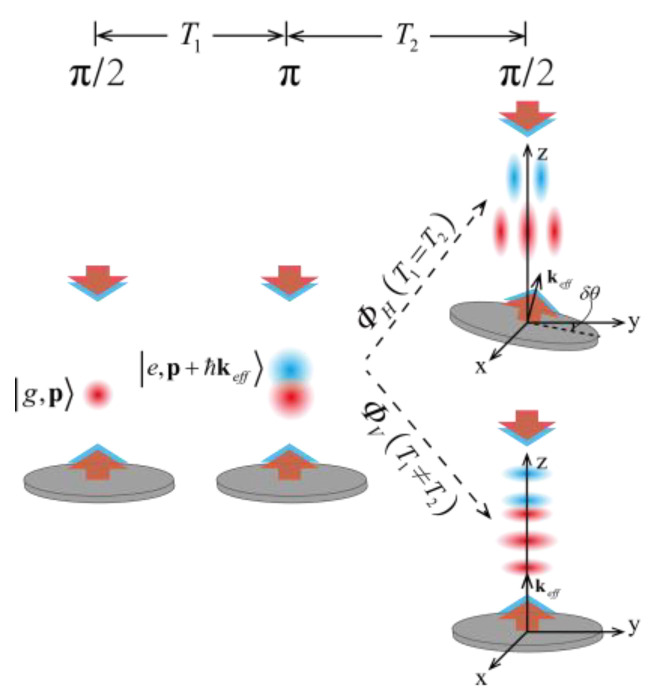
Schematic diagram of phase shears based on interference sequence of π/2−π−π/2 Raman pulses. The two separate branches induce horizontal beam-tilt phase shear $\Phi_{H}$ and vertical timing-asymmetry phase shear $\Phi_{V}$. Red and blue spheres represent the atom cloud in a ground state $|\left.g\right\rangle$ and an excited state $|\left.e\right\rangle$, respectively.

The APSI works in a way similar to its optical counterpart of a shear interferometer. It is the phase shear added to the interferometer that reveals rich details in the total phase shift in an APSI. As is shown in Figure 1, two types of phase shear can be added in an atom interferometer with a π/2−π−π/2 Raman pulse configuration, namely the horizontal beam-tilt phase shear $\Phi_{H}$ and the vertical timing-asymmetry phase shear $\Phi_{V}$. The horizontal phase shear at a specific displacement $y_{3}$, mathematically expressed as $\Phi_{H}=k_{eff}\delta \theta_{x}y_{3}$, is introduced by applying a tilt $\delta \theta_{x}$ to the final π/2 pulse. The vertical timing-asymmetry phase shear along the vertical (z) direction $\Phi_{V}=k_{eff}v_{z}\delta T$ is added by manipulating the asymmetrical timing interval of interferometer pulses, where $v_{z}$ is the velocity of the atom along the z-axis and δT is the difference of the 2 intervals in between 3 Raman pulses.

When point-source approximation ($y\approx v_{y}t_{d}\approx y_{3}t_{d}/t_{3},$ $z=v_{z}t_{d}$) is applied, the derivatives of horizontal and vertical phases can be calculated, and corresponding spatial frequencies of interference fringes are calculated to be:

(3) $$\kappa_{H}=\frac{\partial \Phi_{H}}{\partial y}=k_{eff}\delta \theta \;t_{3}/t_{d},$$

(4) $$\kappa_{V}=\frac{\partial \Phi_{V}}{\partial z}=k_{eff}\delta T/t_{d},$$

where $t_{3}$ denotes the timing when the 3rd Raman pulse is applied, counting from the fountain launch. 

### 2.2. Principal Component Analysis

In actual measurements with an ASPI-type inertial sensor, extraction of interference patterns from a recorded image is itself interfered with by various biases and noises. These extra factors include the envelope the atoms form after free expansion, uneven spatial distribution of detection beams, stray background light, thermal noises in the image sensor, etc. To accurately derive the interference fringes from such an image, an image processing technique based on principal component analysis (PCA) was applied prior to fringe extraction.

The PCA algorithm is a classic data processing method that is commonly used in dimensionality reduction and feature extraction of multidimensional data sets [15]. With this technique, the recorded raw image containing both the interference patterns and disturbances is considered as the superposition of a low rank and a sparse sub-set [16]. The main task of PCA is to derive a new set of variables of the reduced variable dimensions from the original image. In this way, a series of basis vectors which are known as principal components (PC) are obtained. The PCs are orthogonal to each other and span the original set of multi-pixel images. All recorded images can then be broken down to a sum of projections of the original image onto each PC [17]. These PC basis vectors are ranked in descending order according to the relevance between themselves and the original image. The higher-ranked PCs contain more significant features of the input image, and the image has larger projections on these high-ranked PCs.

The process of carrying out PCA is essentially a process of dimensionality reduction and noise reduction. Firstly, the covariance matrix is optimized to obtain its eigenvectors and eigenvalues, which, respectively, represent the PCs and their corresponding variances. Then, different components of the original data set can be obtained using basis transformation. The optimization of the covariance matrix follows two principles. The off-diagonal elements of the covariance matrix are supposed to be as small as possible, because they indicate the correlation between different dimensions. On the contrary, the diagonal elements are supposed to be as large as possible, because they indicate the variance of the corresponding basis vector. Larger variances on most significant PCs would lead to lower residue noise, interfering with the detection.

## 3. Experiment and Methods

**Figure 2:**
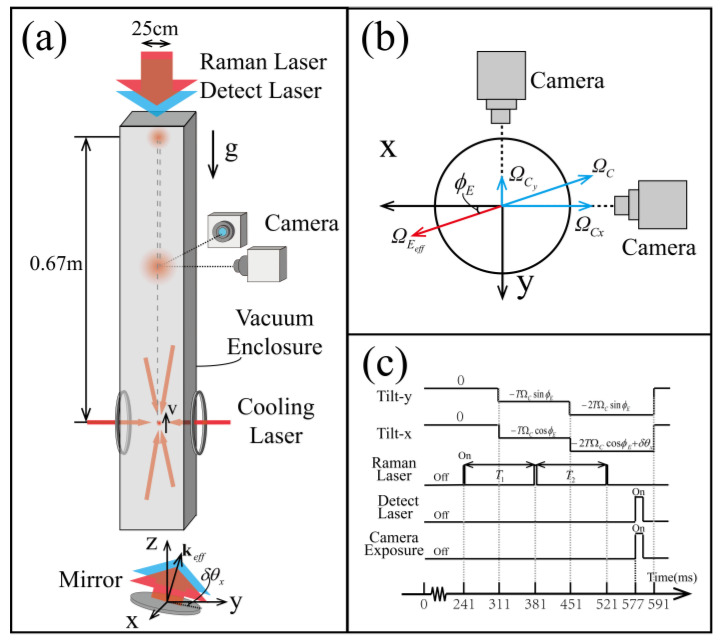
Schematic diagram of the apparatus. (**a**) The schematic diagram of phase shear in the atomic interferometer; (**b**) top view of the Earth rotation compensation stage. (**c**) The sequence of realizing phase shear in the atomic interferometer. The launching of atoms is marked as 0 ms.

The ASPI-type inertia sensor was implemented in an atomic fountain interferometer, as is shown schematically in Figure 2. A combination of a 2-dimensional and a 3-dimensional magneto-optical trap (MOT) captures and cools down ^87^Rb atoms for the fountain. Cooling laser beams are then red-detuned by 12 MHz from the D-line resonance of $\left.\left|5^{2}S_{1/2}F=2\right.\right\rangle \to \left.\left|5^{2}P_{3/2}F’=3\right.\right\rangle$ cooling transition. The loading of cold atoms takes 1 s, and an ensemble of $1.6\times 10^{8}$ ^87^Rb atoms are trapped and cooled for the following fountain process.

When the loading process is finished, the magnetic fields of both 2-D and 3-D MOTs are switched off and atoms are then launched into the interferometer chamber vertically using the moving molasses technique. The 2 pairs of upward and downward cooling laser beams are differentially detuned by ∆ν=3.3 MHz, so that atoms in the MOT region are subject to an upward force along the vertical axis. The atoms are accelerated to 3.63 m/s in about 1.3 ms, enabling them to reach a height of 0.67 m in the fountain.

The atoms are further cooled down when they are in ballistic launch using the polarization gradient cooling technique, where cooling laser beams are detuned far, to Δν=−112 MHz gradually, and the intensity is ramped down to 40% in 2.7 ms. After the cooling process, the atoms reach a temperature of 5 μK and the size of the cloud is about 1 mm. A total of 36 cm above the MOT, a series of microwave pulses and resonant laser pulses pump and purify atoms into a magnetically insensitive Zeeman sub-state of $|\left.5^{2}S_{1/2}F=1,m_{F}=0\right\rangle$ to avoid influences from external magnetic fields on the atoms.

**Figure 3:**
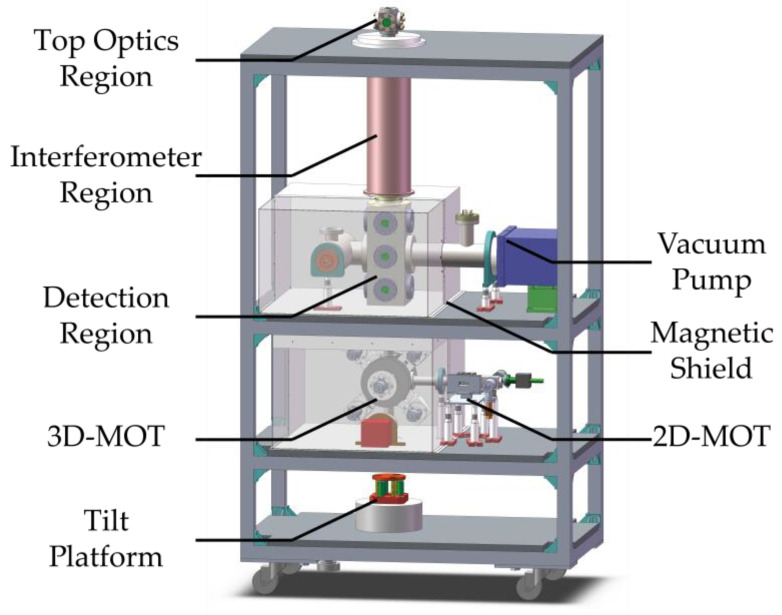
Apparatus of the fountain-type inertia sensor in APSI setup.

Figure 3 shows the actual apparatus of the atom interferometer. The interferometer operates in a Mach-Zehnder setup that uses 3 velocity-sensitive laser pulses to induce stimulated Raman transitions to split, redirect, and recombine the ultra-cold atoms. Time interval between the π/2−π−π/2 Raman pulses is set at *T* = 140 ms. To reduce the influences of non-uniformity of the Raman laser, the light out of a single mode fiber is expanded to diameter of 60 mm, collimated, and then truncated with a 25 mm round diaphragm to match the 25 mm diameter of the vacuum fountain tube. The collimator and diaphragm are mounted on top of the whole instrument and shoot the Raman pulse downward to the bottom, where a mirror is mounted on an electrically controlled tilt stage to reflect it back to top. The phase shear in the APSI setup can then be applied by manipulating the rotation angle or the asymmetrical timing of Raman pulses to induce horizontal or vertical interference fringes.

When the 3-pulse interferometer sequence is finished 0.6 s after fountain launching, a short detection pulse resonant with $\left|5^{2}S_{1/2}F=2\right.\to \left|5^{2}S_{3/2}\left.F=3\right\rangle \right.$ transition is sent from the top of the sensor to the detection region. At the time of detection, the 5 μK atoms have expanded in overall size and occupy a large area in the vacuum chamber. The collimator-diaphragm output port previously used on the Raman laser pulses clamps the pulsed detection beam, leaving only the central part to increase the uniformity of detection. To capture all fringes of interference, a 25×36 mm region at the center of the detection chamber has to be imaged. To fulfil this, an infrared-enhanced lens of 35 mm focal length couples the detection region onto a global-shutter imaging sensor, 20 cm away from center of the chamber and set along the axis of the rotation to be measured. Exposure time of the camera sensor is set at 2 ms to make distortions due to movement of atoms negligible.

It is necessary to compensate for the rotation of the Earth $\Omega_{C}$ to achieve interference patterns sensitive only to the phase shear of the APSI in the laboratory coordinates. An additional rotation of equal magnitude but opposite direction, ${-\Omega }_{C}$, is applied onto the Raman tilting stage. Rough calculation based on the latitude of the lab location gives the direction of the local true north $\phi_{E}$ with respect to the horizon axis *x*. In the measurement, 3 separate tilting steps are applied to the reflecting mirror to achieve the compensation of Earth’s rotation. The tilting angles were set at $-t\Omega_{C}cos\phi_{E}$ and $-t\Omega_{C}sin\phi_{E}$ along the x and y axes, respectively, where *t* is set to 0 at the first Raman pulse for calculation convenience.

**Figure 4:**
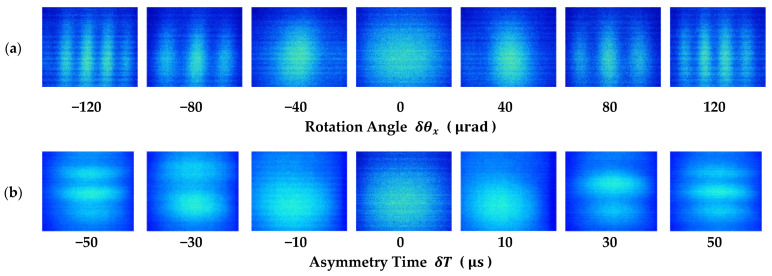
Spatial interference fringes of the atom cloud captured on x-axis camera. (**a**) Horizontal spatial fringes caused by beam-tilt phase shear; (**b**) vertical spatial fringes caused by asymmetrical Raman pulse intervals.

After rough compensation, an extra tilt $\delta \theta_{x}$ is applied to the last π/2 pulse to simulate a small angular velocity $\Omega =2\delta \theta_{x}/t_{3}$ to be measured, which is proportional to the spatial frequency of the horizontal fringes $\kappa_{H}$, as is shown in Equation (3). Figure 4a demonstrates a series of raw captured images with various rotation angles, with expansion time set to be $t_{d}=0.577\;s$, and the last π/2 pulse at $t_{3}=0.521\;s$.

When the Earth’s rotation has been properly compensated, it is also possible to obtain vertical interference fringes by introducing a small asymmetrical timing difference δT into the intervals in between the 3 Raman pulses, namely $T_{1}=T-\delta T/2$ and $T_{2}=T+\delta T/2$. In this case, the fringes are visible on the camera from any horizontal direction. The corresponding images are also shown in Figure 4b with different settings of δT. When phase shear is introduced, readout of the interferometer phase and evaluation of its contrast is feasible within one single measurement cycle.

## 4. Improvement of the Readout of APSIs

The measurement of angular velocity on an APSI relies on the accurate derivation of spatial frequency of the interference fringes captured by the imaging sensor. However, fitting the raw images directly to the theoretical model is far from accurate because of multiple imperfections of the instrument. For example, uneven distribution of atoms’ velocity and, consequently, their displacement from the center of the cloud is always superposed on the spatial fringes supposed to be measured. Thermal noise of the imaging sensor, residue stray light from detection laser pulses, and reflections from the inside of the vacuum chamber also disturb the detection in a way that is difficult to model and predict. To fulfill the measurement, a PCA-based pre-fitting procedure was applied to the detected raw image signals. 

### 4.1. Pre-Fitting Preparation of Images

**Figure 5:**
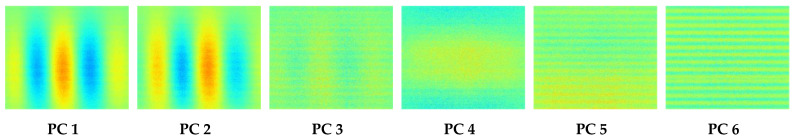
Normalized images of the first 6 principal components obtained from beam-tilt phase shear of $\delta \theta_{x}=60\;\mu rad$.

The PCA-based procedure acts on the detected raw images much like an image filter separating the desired interference patterns from unwanted background bias and noise. As an unsupervised machine learning algorithm, PCA requires multiple raw images as a training set to derive the eigenvectors in subspace. In the experiment, a training set comprises 100 images from consecutive detection with identical phase shear settings. Figure 5 shows a series of principal components obtained from an image set of a beam-tilt phase shear experiment, ranked in descending order according to their respective eigenvalues.

In PCA, the corresponding eigenvalue of a specific principal component reflects the proportion of information it contains in the original signal, and the component with a larger eigenvalue is more related to lower-frequency changes in the image [18]. Among the derived principal components shown in Figure 5, the first two principal components contain most of the information of concern on the spatial interference fringes in the image of atom cloud. The third component is mostly related to thermal noise and background bias when illumination on the sensor is weak, and the fourth is related to the overall density distribution of the atom cloud. The lower ranked fifth and sixth components are composed of high-frequency fluctuations caused by the sensor and shutter, and are irrelevant in the extraction of spatial frequencies.

### 4.2. Extraction of the Spatial Frequency of Fringes

When the intensity variance of the detection beam is not taken into account, the ideal spatial distribution of atoms after beam-tilt phase shear should be a superposition of horizontal fringes and a Gaussian number density distribution. The position-dependent probability of finding an atom on $|\left.F=1\right\rangle$ or $|\left.F=2\right\rangle$ states is given by

(5) $$P_{1}(x)=A_{1}e^{-\frac{{\left(x-b\right)}^{2}}{\sigma_{x}^{2}}}[\frac{1}{2}+\frac{c}{2}sin(k_{x}x+\phi )],$$

(6) $$P_{2}(x)=A_{2}e^{-\frac{{\left(x-b\right)}^{2}}{\sigma_{x}^{2}}}[\frac{1}{2}-\frac{c}{2}sin(k_{x}x+\phi )],$$

where $\sigma_{x}$ is the standard deviation of the Gaussian distribution and ϕ and $k_{x}$ are the phase and frequency of the interference fringes. The probabilities $P_{1}$ and $P_{2}$ are orthogonal and differ by half a period. With PCA filtering out influences caused by the overall distribution of the detection beam, the fringes formed by atoms on the $|\left.F=1\right\rangle$ state are rewritten as

(7) $$P_{Fringe}\left(x\right)=A_{l}e^{-\frac{{\left(x-b\right)}^{2}}{\sigma_{x}^{2}}}sin\left(k_{x}x+\phi \right).$$

To find the spatial frequency $k_{x}$, the highest-ranked principal components that contain information of interference patterns are fitted with this function.

**Figure 6:**
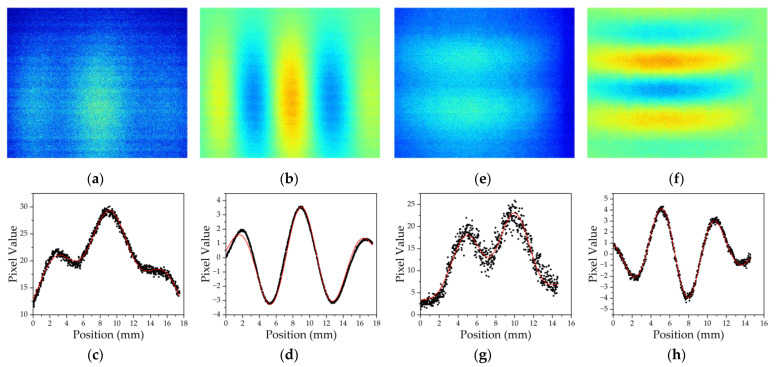
Direct and PCA fitting of spatial fringes. (**a**) and (**e**) are averaged raw images of horizontal and vertical fringe detections; (**b**) and (**f**) are 2nd highest ranked PC of (**a**) and (**e**); lower row of graphs(**c**,**d**,**g**) and (**h**) are integrations of the corresponding image in the upper row, along the direction parallel to fringes. Red lines depict the fitted curve from each data set.

For a comparison of the accuracy and reliability of raw fitting and PCA, both the averaged raw image and the second principal component derived from PCA were integrated along the direction perpendicular to the fringes and subject to parameter fitting according to Formula (7). Figure 6 presents such a comparison for vertical and horizontal interference fringes.

As the PCA-based pre-fitting processing significantly screened out influences via unwanted systematic bias and noises, the characteristics of the interference pattern were significantly enhanced, and the contrast of the fringe as improved by 7–12 dB, resulting in a more reliable recovery of the spatial frequency and phase from atomic interference fringes. The enhancement is effective in both the vertical and horizontal fringe detection schemes. 

### 4.3. Enhanced Readout of APSIs

**Figure 7:**
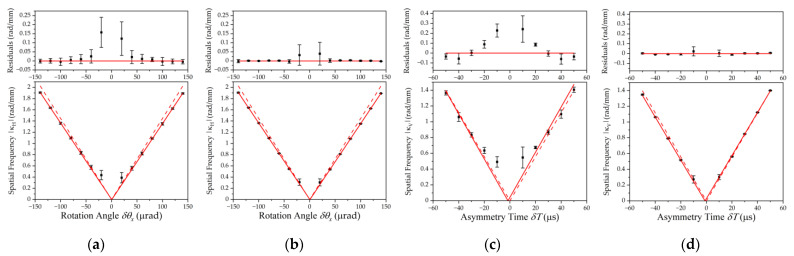
Derived spatial frequency from phase shears. Red solid lines represent theoretical predictions. Red dashed lines represent linear fitting of measured spatial frequencies. (**a**) and (**b**) are results from angular velocity measurements without and with PCA-based pre-fitting processing; (**c**) and (**d**) are results from asymmetrical timing phase shears. Top chart shows the residuals calculated by subtracting the predicted linear values from the measured data.

When the rotation angle in the beam-tilt phase shear scheme or asymmetry time in timing-asymmetry scheme is scanned, spatial frequency extraction with PCA from any image set proves more reliable than that from simple stacking and averaging the images in the set. The comparison is shown in Figure 7. Apart from measurements of very small angles or δt, where the induced interference frequency is so small that less than one period can be detected in the imaging region, the signal-to-noise ratio of spatial fringes after PCA is typically improved by 5–10 dB for beam-tilt phase shear and 9–12 dB for asymmetrical timing phase shear. The enhancement is more significant when the frequency of the induced interference fringes is higher in either setup.

When compared with the theoretical predictions, the improvement by PCA-based fringe enhancement is obvious. In Figure 7, the solid lines in each bottom chart represent the expected linear relationship following Equations (3) and (4), and the difference between measured and expected values is shown in the top charts. When PCA processing is applied prior to frequency acquisitions, the variation of measured spatial frequency is smaller and in better accordance with the predicted linearity. 

In angular velocity measurements with the fountain-type APSI inertia sensor, the precision of the measurement is evaluated by calculating the variation of measured spatial frequency and converting back into rotation velocity following Equation (3). The evaluation gives a precision of 6.3 μrad/s without PCA-based enhancement and 3.3 μrad/s with enhancement from results of the same measurement.

As for the measurement of very small angular velocity or timing asymmetry where the spatial frequency is too low to be precisely recovered from an image in the limited space within the detection chamber, differential measurement by adding an extra rotation angle or timing difference in opposite directions solves the problem, because the small frequency to be measured is transferred to larger frequencies while maintaining the mean value. The PCA-based processing technique is still appliable and works equally well in such differential measurements. However, because these measurements are not as reliable as those of relatively high frequencies in the same measurement scheme, these inaccurate results are ruled out in the evaluation of the enhancement.

## 5. Conclusions

By implementing PCA-based image processing techniques, measurement of angular velocity on an APSI-type inertial sensor is enhanced. A comparison of measurement results with and without the PCA procedure, and the theoretical predictions according to the dependency of the spatial frequency of interference fringes on angular velocity proves the PCA-based technique capable of improving both the accuracy and precision of measurements. The technique is versatile in various applications of atomic interferometers where the detection is carried out via the imaging of fluorescence from stimulated atoms. 

## Data Availability

Data underlying the results presented in this paper are not publicly available at this time, but may be obtained from the authors upon request.
